# CD147 promotes cell motility via upregulation of p190-B RhoGAP in hepatocellular carcinoma

**DOI:** 10.1186/s12935-016-0344-z

**Published:** 2016-09-06

**Authors:** Ruo Chen, Shi-Jie Wang, Yang Zhang, Rong Hou, Jian-Li Jiang, Hong-Yong Cui

**Affiliations:** 1 Department of Cell Biology, National Translational Science Center for Molecular Medicine, Fourth Military Medical University, Xi’an, 710032 People’s Republic of China; 2 Department of Cell Biology, College of Life Science and Technology, Jinan University, Guangzhou, 510632 People’s Republic of China

**Keywords:** CD147, Cell movement, Hepatocellular carcinoma, p190-B RhoGAP, RhoA

## Abstract

**Background:**

The acquisition of inappropriate migratory feature is crucial for tumor metastasis. Rho-family GTPases including RhoA are molecular switches that play critical roles in regulating cell movement. We investigated the molecular mechanism underlying CD147 induced RhoA deactivation in hepatocellular carcinoma (HCC) cells.

**Methods:**

Wound-healing assay was performed to study the cell motility. Analysis of RhoA activation in living cells was conducted using RhoA biosensor. Changes in the expression of certain genes were determined by quantitative real-time PCR. The expression of proteins was evaluated by Western blot. Cytoskeleton reorganization and focal adhesion formation were observed by immunofluorescence staining. Further investigation on the correlation between CD147 and p190-B RhoGAP (p190-B) in HCC tissues was performed by immunological histological chemistry analysis.

**Results:**

CD147 promoted cell movement and suppressed RhoA activation. p190-B, a negative regulator of RhoA activity, was upregulated by CD147 at both mRNA and protein levels. This regulatory relationship was further confirmed by analyzing the expression pattern of CD147 and p190-B in human HCC tissues. Silencing of p190-B caused the increased formation of stress fiber and focal adhesion and blunted the impact of CD147 overexpression on cell movement, indicating that the regulatory effect of CD147 on cell movement is mediated, at least partially, by p190-B.

**Conclusions:**

These findings indicated that p190-B, a negative regulator of RhoA, is positively regulated by CD147 and contributes to the regulation of cell movement in HCC. CD147 plays critical roles in the motility of cancer cells and may be therefore a valuable drug target for anti-cancer therapy.

## Background

The majority of deaths associated with cancer are due to the metastasis of the original tumor cells [1]. The acquisition of inappropriate migratory and invasive characteristics is a common feature of all metastatic cancer cells. Rho family GTPases are intracellular signaling molecules that play critical roles in regulating cytoskeleton reorganization and cell movement. The activities of most Rho family members including RhoA depend on a delicate balance between the GTP-bound, active state and the GDP-bound, inactive state. The cycling between these two states is positively controlled by guanine exchange factors (GEFs), and negatively controlled by GTPase-activating proteins (GAPs) [2]. In the activated form, they are competent in binding to a large number of effector proteins, which leads to the activation of myriad numerous downstream signals. >80 RhoGEFs and >70 RhoGAPs have been discovered to regulate the activities of RhoGTPases in mammals [3, 4]. The overabundance of RhoGEFs and RhoGTPases versus Rho GTPases substrates allows the regulators to function in a tissue/cell type-specific manner [5].

CD147 also known as extracellular matrix metalloproteinase inducer (EMMPRIN), is a type I transmembrane glycoprotein. Previous studies showed that CD147 plays important roles in cellular processes of HCC progression, including adhesion [6], migration [7–9], invasion and metastasis [10]. Of note, CD147 interacts directly with annexin A2 and suppresses RhoA activity [7]. Also, CD147 enhances Src activity and promotes mesenchymal-type cell movement by up-regulating RacGEF DOCK8 [8], suggesting that CD147 may regulate cell motility via downregulation of RhoA and upregulation of Rac1, however, the underlying mechanisms are far from clear.

p190-B RhoGAP (p190-B) is a 190 kDa multidomain protein. The N-terminal domain of p190-B contains several motifs characteristic of a GTPase domain, while its C terminus contains a Rho GAP domain. p190-B is among the most important regulators of RhoA and the actin cytoskeleton. Mice lacking p190-B display defects in cell size, thymus and lung during fetal development. Also, p190-B loss in embryonic mesenchymal/fibroblasts leads to an imbalance in adipogenesis/myogenesis cell fate determination [11–13]. Loss of p190-B enhances hematopoietic stem cell (HSC) self-renewal ability [14] and p190-B is critical for the constitution of a functional mesenchymal-derived hematopoietic niche [15]. In cells, p190-B co-localizes with the α5β1 integrin receptor for fibronectin and is thought to be a negative regulator of RhoA activity [16, 17]. The junctional localization of p190-B can be inhibited by centralspindlin, leading to increased RhoA activity during cytokinesis in interphase cells at the zonula adherens [18]. All these results suggest that p190-B functions mainly via regulating RhoA activity. We hypothesized that if p190-B is critical for promoting RhoA deactivation it might also play an important role during cancer cell movement in HCC.

In this study, we provide evidence that CD147 promotes cell movement while inhibiting RhoA activity. CD147 enhances p190-B expression at both mRNA and protein levels and increased p190-B results in reduced RhoA activation and upregulation of cell movement in HCC cells.

## Methods

### Cell lines and transfection

Human SMMC-7721 hepatoma cell line was obtained from Chinese Academy of Medical Sciences. Huh-7 cells were obtained from the Japanese Collection of Research Bioresources. HepG2 cells were obtained from the American Type Culture Collection. All cell lines were routinely cultured using standard protocols. Transient transfection of plasmids was performed using Lipofectamine 2000 (Life Technologies, US) according to the manufacturer’s protocol.

### Tissue specimens and immunological histological chemistry (IHC) analysis

Forty-seven tissue specimens of HCC were collected from the Department of Hepatobiliary & Pancreas Surgery, Xijing Hospital, which is affiliated with the Fourth Military Medical University (FMMU) from 2008 to 2009 and were histological confirmed by staining with hematoxylin and eosin (H and E). All individuals provided written informed consent, and the study was approved by the hospital Ethics Committee. For IHC analysis, sections were incubated with primary antibodies and developed with the Histostain^®^-Plus Kit (Invitrogen, Carlsbad, CA). The expression level was independently evaluated by two senior pathologists according to the proportion and intensity of positive cells. The following criteria was used to score each specimen: 0 (no staining), 1 (any percentage with weak intensity or <30 % with strong intensity), 2 (>30 % with strong intensity).

### Antibodies, plasmids and biosensor

CD147-specific antibody HAb18 was produced by our lab; α-tubulin antibody (sc-8035) was purchased from Santa Cruz; p190-A antibody (2513) was obtained from Cell Signaling Technology; p190-B antibody (611612) and paxillin antibody (610620) were obtained from BD biosciences; Alexa 594-conjugated goat anti-mouse IgG and Alexa Fluor 488 phalloidin were purchased from Invitrogen. A siRNA-resistant CD147 replacement vector was generated based on a previously reported CD147 expression plasmid [19] using the following primer: forward 5′-TCATGAACGGCTCCGAGAGCAGATTTTTTGTTTCATCCTCGCAGGGCCGGTCAGAGC-3′, reverse 5′-GCTCTGACCGGCCCTGCGAGGATGAAACAAAAAATCTGCTCTCGGAGCCGTTCATGA-3′. The RhoA biosensor was obtained from Addgene.

### Western blot

Cell pellets were lysed with RIPA buffer (Beyotime, China) containing protease inhibitor (Roche, Switzerland). Blots were probed with the appropriate antibodies and developed using ECL kit (Pierce, US).

### RNA interference

Cells were transfected with a pool of siRNAs using HiPerFect Transfection Reagent according to the manufacturer’s instruction (Qiagen). siRNA mixes targeting CD147 or p190-B were designed and synthesized by Shanghai GenePharma Co. (Shanghai, China). Silencer negative control siRNA (siCtrl) was used as a negative control under similar conditions. The sequence for siRNA targeting CD147 used in rescue experiment was 5′-GUUCUUCGUGAGUUCCUCtt-3′.

### Quantitative real-time PCR analysis

Total RNA was extracted using the TRIzol reagents (OMEGA Bio-Tek). Reverse transcription was performed using the PrimeScript RT reagent Kit (TaKaRa Biotechnology). All primers were synthesized by BGI (BGI, Shenzhen, China). Primer sequences were listed in Table X. Real-time PCR was performed using the SYBR Premix Ex Taq II Kit (TaKaRa Biotechnology).

### In vitro wound-healing assay

In vitro wound-healing assay was performed as described previously [8]. Briefly, 24 h after treatment, the cells were harvested and seeded in 12-well plates until confluent. A pipette tip was used to scratch the monolayer. The cells were then washed with serum-free medium. Photomicrographs were obtained at two time points (0 and 24 h), and the relative migration distance was calculated using the following formula: the relative migration distance (%) = 100 (AX−BX)/(A blank−B blank), where A is the width of the cell wound before incubation, and B is the width of the cell wound after incubation.

### Fluorometric analysis of the RhoA activation

Cells were prepared as previously reported [20]. Images were obtained using an A1 confocal microscopy (Nikon, Japan). For emission ratio imaging, the filter sets were used as previously reported [21]. NIS-Elements software (Nikon, Japan) was used to analyze the images following previously described methods [20]. Briefly, images were dark-current and background-subtracted. Binary masks generated through intensity thresholding were applied to each emission channels, and the matched FRET and donor image sets were ratioed to depict RhoA activation throughout the cell. A linear pseudocolour lookup table was applied.

### Immunofluorescence

Immunofluorescence was performed as described previously [8]. Briefly, cells were harvested and allowed to attach for 24 h to fibronectin-pre-coated cell culture dishes with glass bottoms (801002, NEST Biotechnology Co., LTD.). After washing twice with PBS, the cells were fixed in paraformaldehyde in PBS, permeabilized with 0.1 % Triton X-100, and blocked with 1 % BSA in PBS for 1 h. The dishes were first incubated with the indicated antibodies for 1 h, washed twice with PBS, and then incubated with Alexa 488-phalloidin solution and the corresponding FITC-conjugated secondary antibodies for 30 min in the dark. Cell nuclei were dyed with DAPI (Vector Labs). After washing, the cells were visualized using an A1R-A1 confocal laser microscope system (Nikon, Japan).

### Statistical analysis

The results were expressed as the mean value ± SD of individual experiments. Comparisons of means were conducted using student’s t test or one-way ANOVA (GraphPad Prism, version 6.0). Spearman correlation analysis was used to assess the correlation between CD147 and p190-B in tissues. P values less than 0.05 were considered as statistically significant.

## Results

### CD147 promotes cell movement and inhibits RhoA activation

**Fig. 1:**
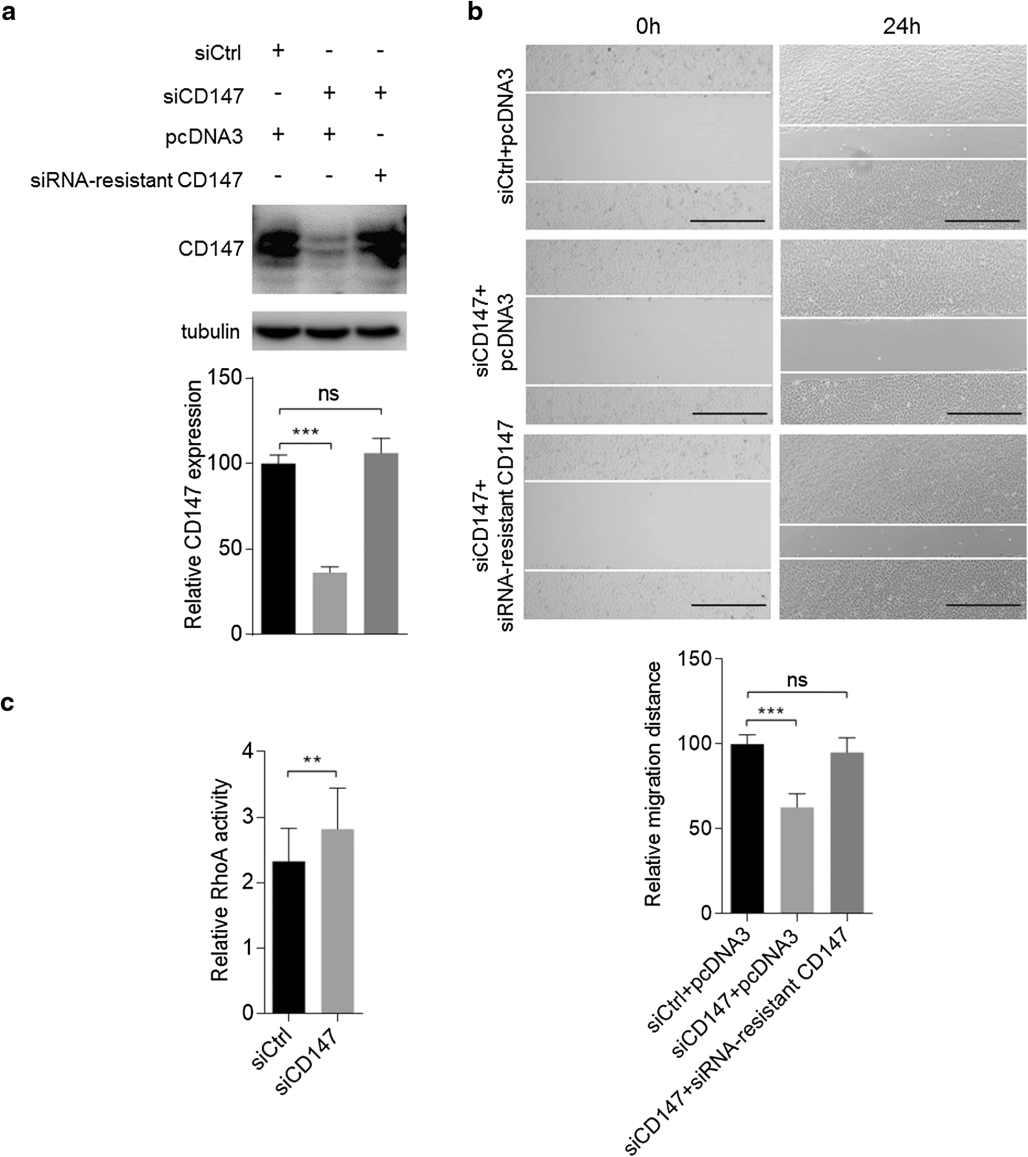
CD147 promotes cell movement and inhibits RhoA activation. **a** The expression of CD147 was measured by Western blot at 48 h after transfection with the indicated constructs. **b** Quantitative analysis of relative migration distance of SMMC-7721 cells transfected with the indicated constructs. **c** Quantification of RhoA activation at 48 h after transfection. **p < 0.01, ***p < 0.001 by student’s t test. *Error bars* indicate the standard deviations (SD) from at least triplicate determinations

We began by determining the effects of CD147 silencing on HCC cells motility. The expression of CD147 in cells transfected with siRNAs targeting CD147 (siCD147) was significantly reduced compared to the cells transfected with control siRNA (siCtrl) (Fig. 1a). Wound-healing assay was used to investigate the role of CD147 in cell movement in HCC cells. A significant decrease was observed in SMMC-7721 cells transfected with siCD147 compared to the control cells (Fig. 1b). To exclude the possibility of off-target effect of siRNA-mediated CD147 interference, a siRNA-resistant CD147 replacement vector was generated. The expression of CD147 in cells cotransfected with siCD147 and the CD147 replacement vector was rescued (Fig. 1a) and cell motility was also rescued (Fig. 1b), indicating that CD147 exerts positive regulation over hepatoma cells. RhoA, one of the most extensively studied members of the Rho family of small GTPases, is most readily recognized for its contributions to actin-myosin contractility and stress fiber formation mainly by activating effector proteins such as Rho kinase, which promotes myosin-2 activation [22]. Thus, we determined the effects of CD147 silencing on RhoA activation. We found that CD147 silencing led to increased RhoA activation (Fig. 1c), indicating that CD147 may regulate cell movement via inhibiting RhoA activation.

### CD147 promotes p190-B expression in HCC cells

**Fig. 2:**
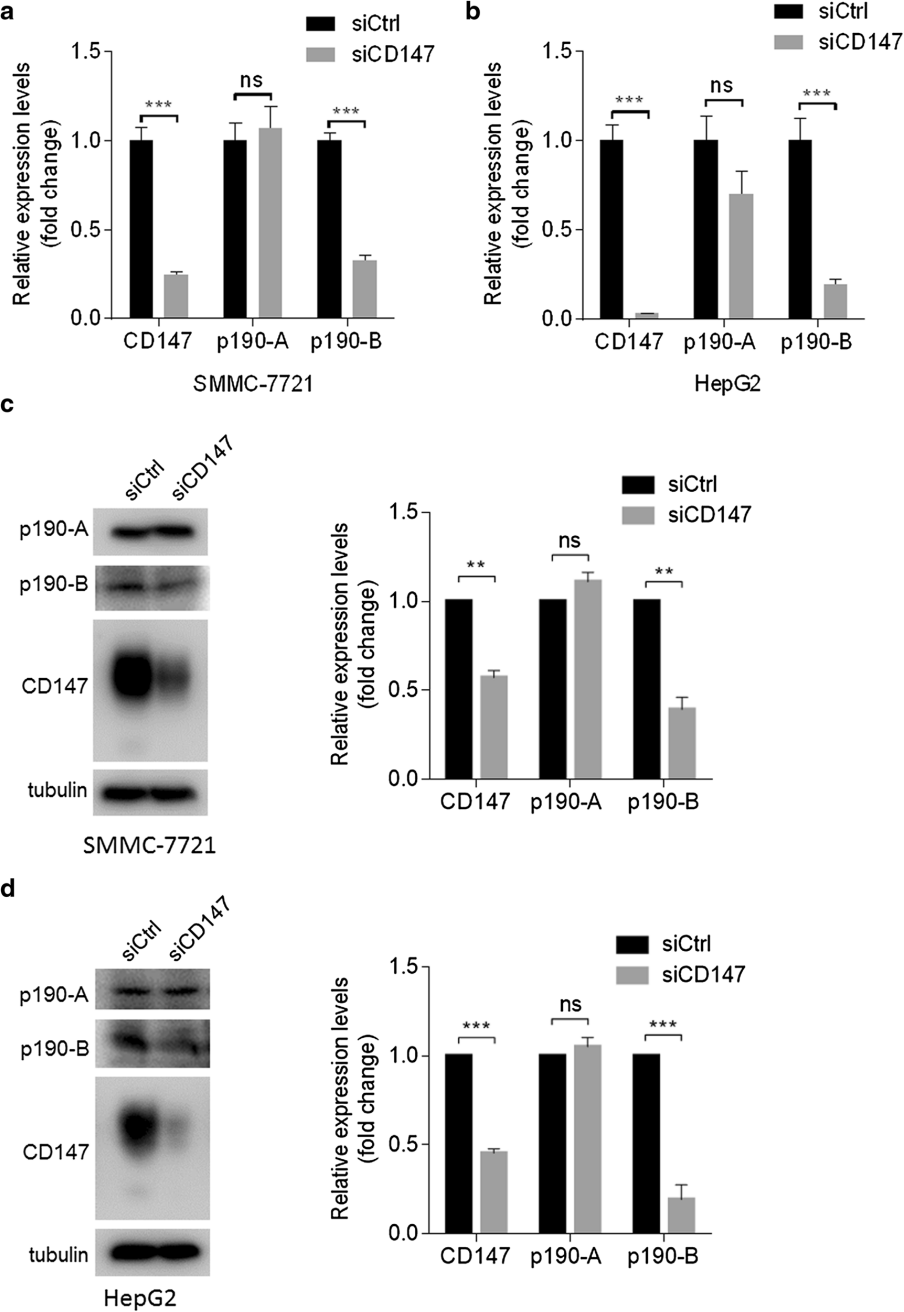
Silencing CD147 leads to decreased p190-B expression. **a**, **b** Relative quantitative real-time RT-PCR analysis of p190 RhoGAP family in indicated cells transfected with siCD147 or siCtrl at 24 h after transfection. ***p < 0.001, ns p > 0.05 by student’s t test. **c**, **d** The expression of p190-A or p190-B in indicated cells transfected with siCD147 or siCtrl was determined by Western blot at 48 h after transfection

**Fig. 3:**
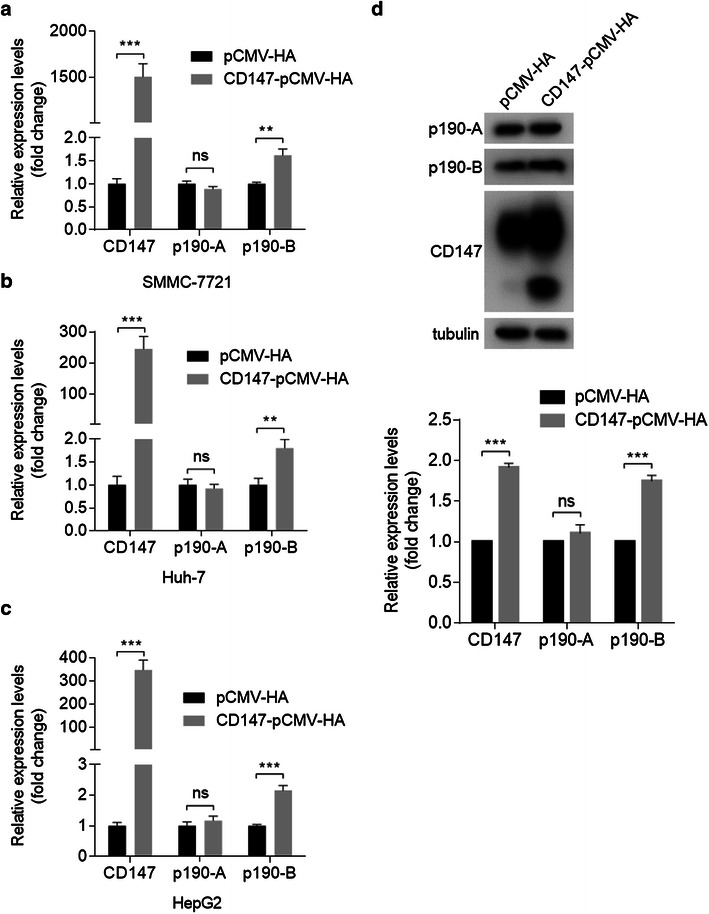
Overexpression of CD147 results in upregulation of p190-B expression. **a**–**c** Relative quantitative real-time RT-PCR analysis of p190 RhoGAP family in indicated cells transfected with pCMV-HA or CD147-pCMV-HA at 24 h after transfection. ***p < 0.001, **p < 0.01, ns p > 0.05 by student’s t test. **d** The expression of indicated molecules in SMMC-7721 cells transfected with pCMV-HA or CD147-pCMV-HA was determined by Western blot at 48 h after transfection. *Error bars* indicate SD from at least triplicate determinations

To determine the mechanism underlying the downregulation of RhoA activation by CD147 silencing, we examined the expression of p190 RhoGAPs. Strikingly, we found that p190-B, but not p190-A, was decreased in HCC cells transfected with siCD147 both at mRNA (Fig. 2a, b) and protein levels (Fig. 2c, d). Also, forced expression of CD147 in HCC cells resulted in increased expression of p190-B both at mRNA (Fig. 3a, c) and protein levels (Fig. 3d), indicating that CD147 promotes p190-B expression both at mRNA and protein levels in HCC cells.

### p190-B expression is positively correlated with the expression of CD147 in HCC tissues

**Fig. 4:**
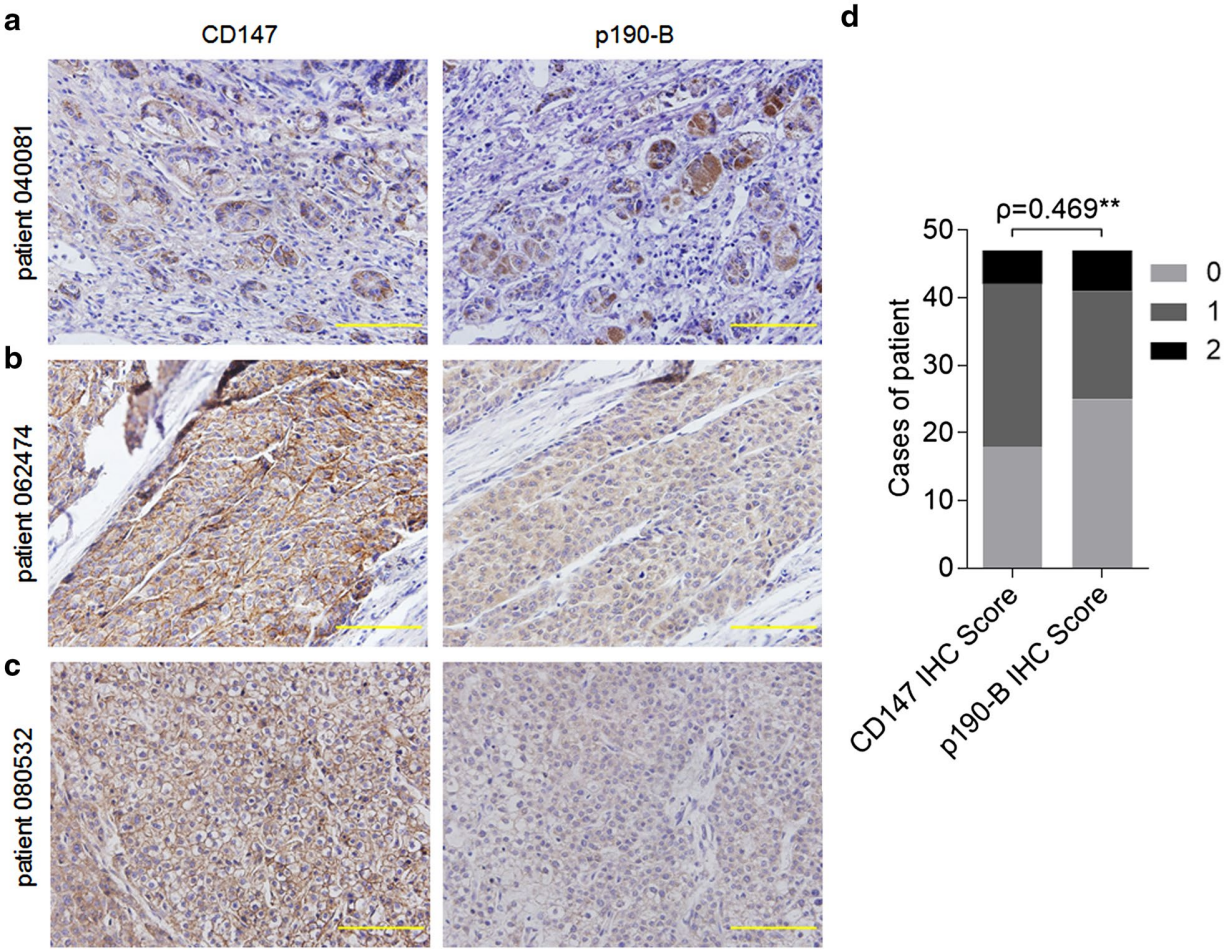
Immunohistochemical analysis of CD147 and p190-B expression in the resected tumor. **a**–**c** Representative images of immunohistochemical staining for CD147 and p190-B in paired HCC tissues. The scores for CD147 in patient 040081, 062474 and 080532 were grade 1, grade 2 and grade 2, respectively. The scores for p190-B in the indicated patients were grade 2, grade 2 and grade 1, respectively. The *scale bar* represents 10 μm. **d** The relationship between CD147 and p190-B expression. **p = 0.001 by Spearman correlation

To further illuminate the regulatory role of CD147 in p190-B expression, the expression of CD147 and p190-B in HCC tissues was evaluated by IHC. As shown in Fig. 4a–c and Table 1, most samples exhibited similar expression patterns of CD147 and p190-B. Spearman correlation analysis based on IHC score indicated a significant positive correlation between the expression levels of the two proteins (Fig. 4d). Together, these findings identify p190-B as a major RhoGAP regulated by CD147 in HCC.

**Table 1:** Correlation between CD147 and p190-B expression in HCC tissues

CD147 expression	p190-B expression			Total, *N*
	–	+	++	
–	15	3	0	18
+	8	12	4	24
++	2	1	2	5
Total, *N*	25	16	6	47

Using the Spearman correlation analysis, ρ = 0.469, p = 0.001

### p190-B promotes cell movement and inhibits RhoA activation

**Fig. 5:**
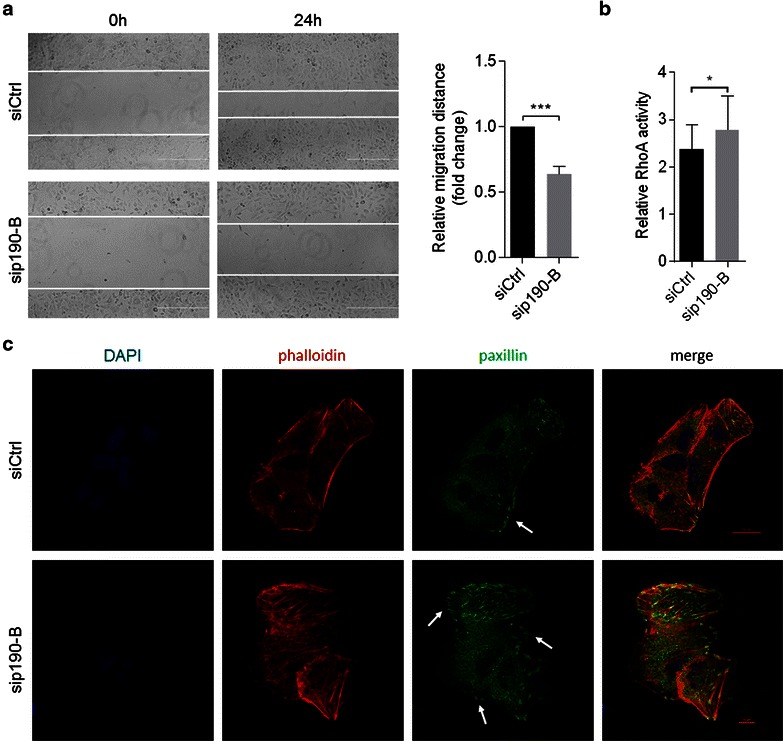
p190-B promotes cell movement and inhibits RhoA activation. SMMC-7721 cells were transfected with a pool of siRNAs targeting p190-B or siCtrl. **a** Quantitative analysis of relative migration distance by wound-healing assay. ***p < 0.001 by student’s t test. **b** Quantification of RhoA activation in indicated cells at 48 h after transfection. *p < 0.05 by student’s t test. **c** Cells were stained with DAPI, rhodamine-conjugated phalloidin and an antibody against paxillin and imaged by confocal microscopy. *Arrows* indicate focal adhesions. *Error bars* indicate SD from at least triplicate determinations

To better understand the role of p190-B in cell movement, we determined the effects of p190-B silencing on cell movement and RhoA activation in HCC cells. We found that p190-B silencing resulted in decreased cell movement (Fig. 5a) and increased RhoA activation (Fig. 5b), indicating that p190-B may regulate cell movement via inhibiting RhoA activation. As RhoA regulates cell movement via stimulating cytoskeleton rearrangement, we examined whether p190-B could affect stress fiber distribution and focal adhesion formation. We found that p190-B silencing resulted in increased stress fiber and focal adhesion (Fig. 5c).

### CD147 promotes cell movement by enhancing p190-B expression

**Fig. 6:**
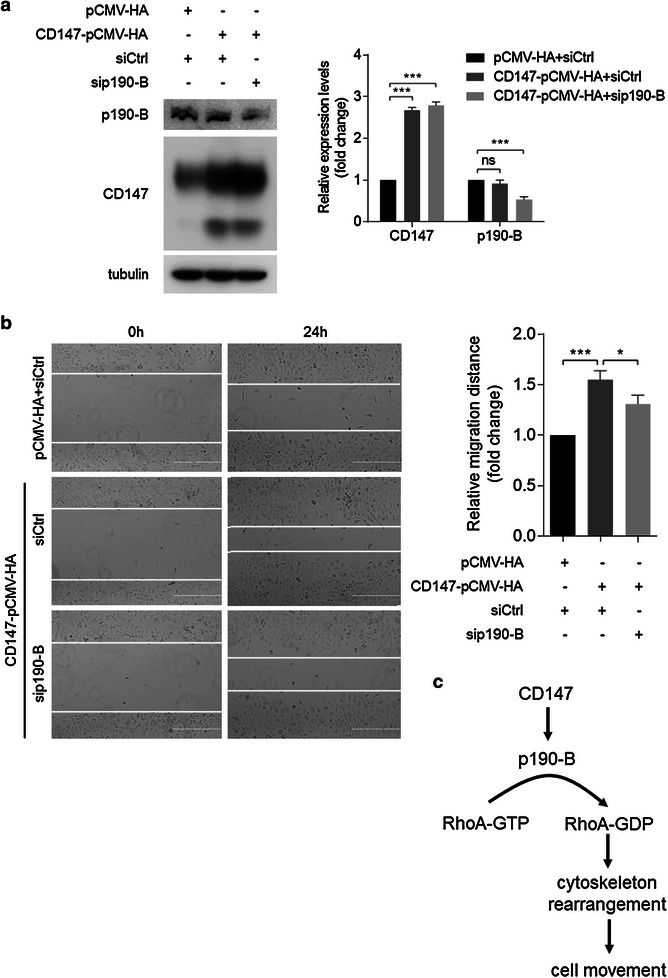
CD147 inhibits RhoA activation through enhancing p190-B expression. SMMC-7721 cells were transfected with the indicated constructs. **a** The expression of CD147 and p190-B was analyzed by Western blot. **b** Quantitative analysis of relative migration distance by wound-healing assay. *p < 0.05, ***p < 0.001 by ANOVA. **c** Schematic representation of major mechanisms of CD147 in regulating cancer cell movement via promoting p190-B expression. *Error bars* indicate SD from at least triplicate determinations

We have demonstrated that CD147 promotes cell movement and p190-B expression. We next tried to investigate whether p190-B-mediated RhoA deactivation was involved in the regulation of cell movement by CD147. This hypothesis predicted that reducing p190-B should impair the impact of CD147 overexpression on cell motility and RhoA deactivation. Accordingly, we transfected SMMC-7721 cells with CD147-pCMV-HA, and then selectively depleted p190-B by a pool of siRNAs (Fig. 6a). Cell motility was increased in cells transfected with CD147-pCMV-HA together with control siRNAs. Strikingly, however, CD147 overexpression did not enhance cell motility to the same level in p190-B knockdown cells as it did in control cells (Fig. 6b). This implies that p190-B contributes to the regulation of cell movement by CD147.

## Discussion

In this study we found that CD147 promotes p190-B expression both at mRNA and protein levels. p190-B was further identified as a RhoA GAP and was able to inhibit RhoA activation and to promote cell movement in HCC cells. Also, we demonstrated that the regulation of RhoA activation and cell movement by CD147 was mediated, at least partially, by p190-B in HCC (Fig. 6c), indicating that RhoA is an indirect target of CD147.

Members of the Rho GTPase family including RhoA operate as molecular switches that affect downstream signaling pathways and play a critical role in cytoskeleton dynamics and cell migration [23]. p190-B is a negative regulator of RhoA activity [16], which is consistent with our finding reported here. Aside from the canonical Rho GTPase RhoA, three isoforms have been identified. RhoA, RhoB and RhoC have high amino acid sequence homology. However, they differ in many biochemical characteristics and cellular functions. The specificity of p190B and the role of RhoB and RhoC in CD147-regulated cell movement are still far from clear. Overexpression of p190-B in MCF-10A cells results in reduced actin stress fiber network and exhibit circumferential staining for actin [24] and knockdown of p190-B decreases cell spread and migration in Huh-7 cells [17], suggesting a role for p190-B in regulating the signaling pathways that influence cancer cell migration and invasion. In vivo analysis using transgenic mice indicates that p190-B has pro-tumorigenic functions that enhance MMTV-Neu induced mammary tumor formation and metastasis [25]. Here, we show that siRNA-mediated silencing of p190-B in HCC cells causes reduced RhoA activation and increases formation of stress fiber and focal adhesion, leading to decreased cell movement. Furthermore, CD147, a key regulator of HCC progression, is able to promote p190-B expression both at mRNA and protein levels and exerts positive influence on cancer cell motility mediated at least partially by p190-B. This regulatory relationship was further verified by immunohistochemistry staining of CD147 and p190-B in HCC tissues. Therefore, p190-B is involved in the regulation of cell movement by CD147 as a major GAP for RhoA in HCC cells.

CD147 has been reported to play important roles in HCC progression, including migration and invasion [10, 19, 26]. Of note, CD147 interacts with integrin β1, enhances expression and phosphorylation of FAK and paxillin, and subsequently leads to cytoskeletal rearrangement and morphological changes [27, 28]. Also, it is believed that CD147 plays a role in mediating epithelial-mesenchymal transition (EMT) in the process of HCC progression, providing a slight clue to the function of CD147 in cytoskeleton rearrangement [29, 30]. Recently, we reported that CD147 promotes cell motility by regulating Annexin A2-activated RhoA [7], enhances Src activity and promotes mesenchymal-type cell movement by up-regulating RacGEF DOCK8 [8], is able to promote WAVE2-mediated lamellipodia formation and cell movement [9]. All these results, together with our data reported here, suggest that CD147 regulates cytoskeleton rearrangement and cell movement at multiple levels and through different mechanisms, however, how to coordinate these mechanisms during regulating cell movement still awaits further investigation and it is still difficult to dissect to what extent each mechanism contributes to regulation of cell movement under diverse circumstances. Above all, targeting CD147 could be a promising strategy to reduce migration and metastasis of tumor cells.

## Conclusions

The results of this study do support the regulatory role of CD147 on cytoskeleton rearrangement in HCC. p190-B, a negative regulator of RhoA, contributes to the regulation of RhoA activation and cell movement by CD147. CD147 could be a promising target molecule for developing new anti-cancer drugs.

## Abbreviations

p190-B: p190-B GTPase activating protein
HCC: hepatocellular carcinoma
RhoA: ras homolog gene family, member A
GEF: guanine exchange factor
GAP: GTPase-activating protein
DOCK: dedicator of cytokinesis
HSC: hematopoietic stem cell
IHC: immunological histological chemistry
GAPDH: glyceraldehyde 3-phosphate dehydrogenase
FRET: fluorescence resonance energy transfer
FITC: fluorescein isothiocyanate
DAPI: 4′,6-diamidino-2-phenylindole
FAK: focal adhesion kinase
EMT: epithelial-mesenchymal transition
siRNA: small interfering RNA
SD: standard deviation
ANOVA: analysis of variance
